# Validity of six prognostic scoring systems for septic shock patients admitted to a medical ICU

**DOI:** 10.1186/cc11023

**Published:** 2012-03-20

**Authors:** B Khwannimit, R Bhurayanontachai

**Affiliations:** 1 Prince of Songkla University, Hat Yai, Thailand

## Introduction

This study aimed to assess the validity of the APACHE II, SAPS II, and SAPS 3, along with each of their customized scores, in predicting hospital mortality in patients with septic shock admitted to our ICU.

## Methods

A prospective cohort study was conducted over a 6-year period in a medical ICU of a tertiary referral university teaching hospital in Thailand.

## Results

**Figure 1:**
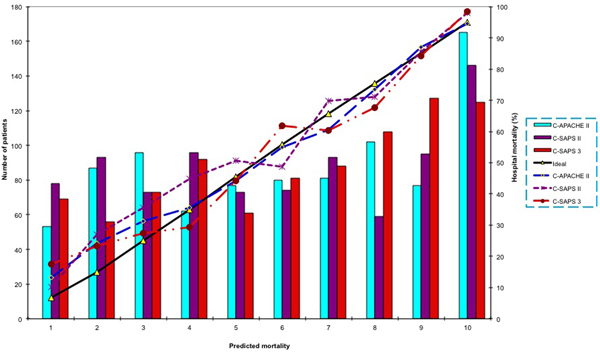
**Calibration curves for customized APACHE II, SAPS II and SAPS 3**.

A total of 880 patients were enrolled and a hospital mortality rate of 57.4% was found. Community-acquired infections accounted for 57.2% and 32.8% of patients had positive blood culture. The respiratory tract was the most common site of infection (48.7%). The predicted mortality of all the scores was close to the observed mortality, with a standardized mortality ratio (95% confidence interval) of 0.94 (0.86 to 1.02) for APACHE II, 1.01 (0.92 to 1.1) for customized APACHE II, 0.93 (0.85 to 1.01) for SAPS II, 1.07 (0.98 to 1.17) for customized SAPS II, 0.97 (0.89 to 1.06) for SAPS 3 and 1.02 (0.93 to 1.11) for customized SAPS 3. All six scores were well discriminated, with areas under the receiver operating characteristic curves of 0.82, 0.813, 0.819, 0.815, 0.817 and 0.813, respectively. The Hosmer-Lemeshow goodness-of-fit showed good calibration in only the customized APACHE II (*H*-statistic 12.4, *P *= 0.26). See Figure 1.

## Conclusion

In this study, the customized APACHE II was found to be accurate in predicting hospital mortality in septic shock patients requiring ICU admission.

